# Patient Experience and Feedback After Using an Electronic Health Record–Integrated COVID-19 Symptom Checker: Survey Study

**DOI:** 10.2196/40064

**Published:** 2022-09-13

**Authors:** Andrew W Liu, Anobel Y Odisho, William Brown III, Ralph Gonzales, Aaron B Neinstein, Timothy J Judson

**Affiliations:** 1 Center for Digital Health Innovation University of California, San Francisco San Francisco, CA United States; 2 Department of Urology University of California, San Francisco San Francisco, CA United States; 3 Department of Medicine University of California, San Francisco San Francisco, CA United States; 4 Bakar Computational Health Sciences Institute University of California, San Francisco San Francisco, CA United States; 5 Clinical Innovation Center University of California, San Francisco San Francisco, CA United States; 6 Office of Population Health University of California, San Francisco San Francisco, CA United States

**Keywords:** COVID-19, patient portals, digital health, diagnostic self evaluation, medical informatics, internet, telemedicine, triage, feedback, medical records systems, San Francisco, user experience, user satisfaction, self-triage, symptom checker, health system, workflow, feedback, integration, electronic health record

## Abstract

**Background:**

Symptom checkers have been widely used during the COVID-19 pandemic to alleviate strain on health systems and offer patients a 24-7 self-service triage option. Although studies suggest that users may positively perceive web-based symptom checkers, no studies have quantified user feedback after use of an electronic health record–integrated COVID-19 symptom checker with self-scheduling functionality.

**Objective:**

In this paper, we aimed to understand user experience, user satisfaction, and user-reported alternatives to the use of a COVID-19 symptom checker with self-triage and self-scheduling functionality.

**Methods:**

We launched a patient-portal–based self-triage and self-scheduling tool in March 2020 for patients with COVID-19 symptoms, exposures, or questions. We made an optional, anonymous Qualtrics survey available to patients immediately after they completed the symptom checker.

**Results:**

Between December 16, 2021, and March 28, 2022, there were 395 unique responses to the survey. Overall, the respondents reported high satisfaction across all demographics, with a median rating of 8 out of 10 and 288/395 (47.6%) of the respondents giving a rating of 9 or 10 out of 10. User satisfaction scores were not associated with any demographic factors. The most common user-reported alternatives had the web-based tool not been available were calling the COVID-19 telephone hotline and sending a patient-portal message to their physician for advice. The ability to schedule a test online was the most important symptom checker feature for the respondents. The most common categories of user feedback were regarding other COVID-19 services (eg, telephone hotline), policies, or procedures, and requesting additional features or functionality.

**Conclusions:**

This analysis suggests that COVID-19 symptom checkers with self-triage and self-scheduling functionality may have high overall user satisfaction, regardless of user demographics. By allowing users to self-triage and self-schedule tests and visits, tools such as this may prevent unnecessary calls and messages to clinicians. Individual feedback suggested that the user experience for this type of tool is highly dependent on the organization's operational workflows for COVID-19 testing and care. This study provides insight for the implementation and improvement of COVID-19 symptom checkers to ensure high user satisfaction.

## Introduction

### Background

During the COVID-19 pandemic, symptom checkers have become an essential tool for providing patients with on-demand access to triage recommendations [1-5]. These tools take patients through self-guided questions about demographics, symptoms, exposures, and past medical history and suggest a diagnosis or recommend a disposition. They employ algorithms and automation to connect patients to care without requiring intervention from clinical staff. COVID-19 symptom checkers have a variety of potential benefits. When used for triage, they may reduce the risk of COVID-19 transmission [4,5] and provide patients with care advice more quickly and efficiently than other methods (ie, telephone hotlines) [6,7]. When used for daily entry screening, they greatly reduce the need for staffing to conduct manual screens [8-10].

### Symptom Checkers

Symptoms checkers are widely considered to be popular with patients because they provide them with 24-7 access to health information, risk assessments, and in some cases, test and appointment scheduling. A handful of studies have reported moderately high user satisfaction ratings for COVID-19 symptom checkers. One study of 296 patients who were predominantly health care workers reported 56% found their institution’s internal web-based symptom checker tool useful [11]. Symptom checkers for conditions other than COVID-19 have also reported positive user experiences, including 1 study reporting high satisfaction in 70%-80% of users [9]. In a study of 22 college students, qualitative factors related to the decision to use publicly available symptom checkers included presence and knowledge of COVID-19 symptoms; fear of in-person health care services; awareness, paranoia, and curiosity; technical literacy; and acceptability [10]. Reported negative characteristics of symptom checkers included doubting accuracy, limited symptom submission possibilities, and unclear logic model of symptom checker [10].

While these studies suggest that users may positively perceive web-based symptom checkers, they are insufficient to understand the patient and tool characteristics that contribute to user experience. Furthermore, no studies have quantified user feedback after the use of an electronic health record (EHR)–integrated COVID-19 symptom checker with self-scheduling functionality. Symptom checkers that are EHR-integrated and offer self-scheduling may have higher user satisfaction because they decrease the time it takes for patients to be scheduled for necessary appointments or tests [6].

As symptom checkers become more ubiquitous for COVID-19 and other use cases, it is important to understand user perceptions and to know which features make them attractive. This type of user feedback can inform product development and improvement for these symptom checkers and any other digital health tools. In addition, it is important to understand whether there are demographic differences that drive user perceptions of the tool, as these may impact health disparities.

### Patterns of Symptom Checker Usage

At the University of California, San Francisco (UCSF), in March 2020, we launched one of the first COVID-19 symptom checkers with self-triage and self-scheduling capabilities. We designed a survey to collect feedback and user experience upon use. This is the first known study to conduct user research on an EHR-integrated symptom checker with self-scheduling functionality. Our primary aims in this analysis are to examine patterns in user experience and user satisfaction by demographic characteristics, determine what patients view as alternatives to symptom checker use, and gather actionable feedback for symptom checker improvements.

## Methods

### Setting

UCSF Health is a large academic health system providing approximately 1.7 million outpatient visits annually. The UCSF primary care practices serve approximately 90,000 empaneled patients. As of January 2022, approximately 95% of adult primary care patients were enrolled in UCSF’s EHR-tethered patient portal.

In early March 2020, UCSF established a COVID-19 telephone hotline, which became the primary telephone intake point for all UCSF patient and employee inquiries regarding COVID-19, including general questions, exposures, symptom assessments, and test scheduling requests.

### Symptom Checker Tool

The UCSF COVID-19 Symptom Checker was developed as an EHR-tethered portal-based self-service option for patients with symptoms of or exposure to COVID-19 or those who are requesting a COVID-19 test. After answering a series of branched logic questions about exposures, symptoms, and comorbidities, patients are directed to the appropriate disposition based on their predicted risk level. The triage algorithm used in this tool was identical to the one used on the telephone hotline. UCSF uses a commercially available EHR from Epic Systems. In early March 2020, we used our EHR vendor’s configuration tools to design, configure, and deploy our UCSF COVID-19 Symptom Checker, which launched on March 12, 2020 [6]. Patients could access the tool by logging into the patient portal on a smartphone, tablet, or computer and were directed to the tool from the hotline, primary care phone tree, and UCSF websites. The tool was available in English and Spanish—the two languages currently supported by our patient portal. The Symptom Checker was available to all adult patients at UCSF with active patient portal accounts [1].

If appropriate based on their responses, patients could self-schedule SARS-CoV-2 RNA tests, video visits, or in-person appointments directly through the tool, as described previously [6]. When patients chose to schedule their test or visit online, a scheduling tool opened within the Symptom Checker, displaying available appointments and allowing the patient to select one. If no appointments were available, patients were directed to call the telephone hotline.

### User Feedback Survey

The optional, anonymous user feedback survey for the UCSF COVID-19 Symptom Checker was built on Qualtrics and consisted of 12 total questions (Multimedia Appendix 1). The survey link was embedded in the final screen of the tool, where patients were shown their care recommendation. The survey was optional, and there were no reminders or prompts to promote survey completion. Patients who did not complete the tool were not able to access the survey.

We designed the survey to be lightweight and easy to complete. Questions were a combination of slider-style rating questions, multiple-choice, select-all-that-apply, and optional open-ended questions. Only the first question, in which patients were asked about their overall rating of the tool, was required. The patients were then asked to assess their agreement on Likert scale with statements describing their experience and about how they would have sought care had the tool not been available. Additionally, the participants were asked about the most important features to them and were able to comment on any technical difficulties experienced. The respondents were asked optional demographic questions on age, gender, ethnicity, and race.

### Study Population

For this analysis, we included all adults who responded to the Qualtrics feedback survey from December 16, 2021, until March 28, 2022. A subanalysis of the peak of the omicron surge (December 16, 2021, to January 28, 2022) and its effect on patient satisfaction and experience was conducted. We defined the peak as the period during which the tool averaged over 200 unique users a day.

### Ethics Approval

This study was approved by the UCSF Institutional Review Board (20-30903).

### Evaluation and Statistical Analysis

Data were exported from the Qualtrics survey and analyzed using R 3.5.1 (The R Foundation). The participants’ longitude and latitude at the time of response based on Qualtrics estimation using respondent IP addresses were matched to respective census block tract and area deprivation index national percentile as a proxy for socioeconomic status [12]. Descriptive statistics were used to analyze user responses. Differences in the user cohort based on responses were analyzed using the 2-tailed chi-squared test for categorical variables and the two-sample, 2-tailed *t* test for continuous variables. Visualizations were created using the *ggplot2* library. A multivariate linear regression analysis was performed with a primary outcome of user overall rating and the demographic questions of the survey. For the purposes of multivariate analysis, respondent ethnicity was stratified as non-Hispanic and Hispanic, and race was stratified as White and non-White. To assess the collinearity of covariates, variable inflation factors were calculated with a cutoff of <10. Moreover, *P*<.05 was considered significant.

Constructive open-ended responses were assigned 1 of 10 categories by a physician reviewer who was familiar with the ambulatory COVID-19 care structure. We did not categorize responses that were purely complementary or that did not offer specific feedback.

## Results

### Survey Response Data

**Figure 1:**
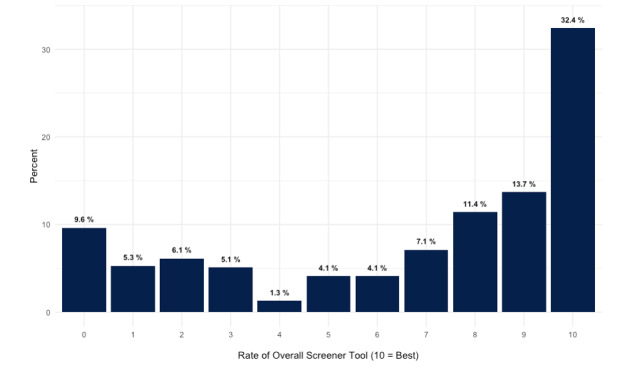
Overall user ratings of COVID-19 Symptom Checker (n=395).

**Figure 2:**
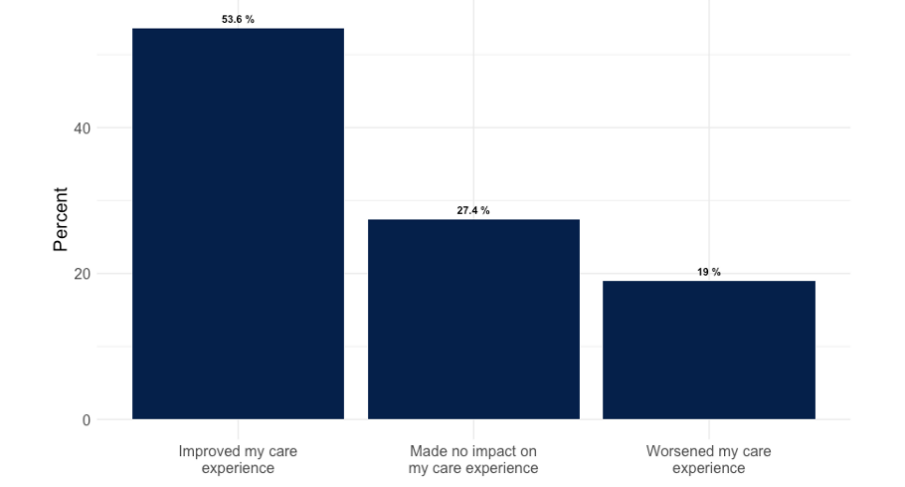
Patient-reported impact of COVID-19 Symptom Checker on care experience (n=332).

From December 16, 2021, until March 28, 2022, there were 395 total responses to the experience survey (Figure 1). During that time, the Symptom Checker was used 29,384 times for a response rate of 1.6%. The median 1-10 rating was 8 (IQR 3-10). In total, 182 users (46.1%) rated their overall experience 9 or 10. When asked how the Symptom Checker affected the overall care experience, about half of the users (n=178, 53.6%) responded that the COVID-19 Testing and Care Tool “improved my care experience,” 91 users (27.4%) responded that the tool “made no impact on my care experience,” and 63 users (19.0%) said the tool “worsened my care experience” (Figure 2). Most users strongly agreed (162/332, 48.8%) or agreed (44/332, 13.3%) that the tool “helped them get the care I needed,” while 23.1% (77/332) strongly disagreed.

**Figure 3:**
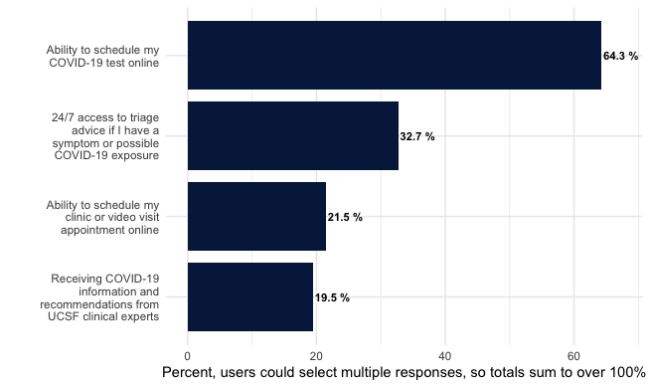
Most important COVID-19 Symptom-Checker features (n=395). UCSF: University of California, San Francisco.

When asked about the most important feature of the tool, over half of the users (n=254, 64.3%) cited the ability to schedule their COVID-19 test online. The second most popular feature was 24-7 access to triage advice if they had COVID-19 symptoms or exposure (n=129, 32.6%; Figure 3). Most respondents (253/331, 76.4%) reported no technical difficulties while using the tool. The most commonly reported technical difficulty was problems with visit or test scheduling. When asked about usability, 52.4% (174/332) of users strongly agreed with the statement that “this tool was easy to use,” while 10.2% (34/332) of respondents strongly disagreed. Most respondents (208/332, 62.7%) strongly agreed with the statement that “questions were easy and clear to understand,” while 7.2% (24/332) strongly disagreed.

### Omicron Surge Subanalysis

There were 288 responses during the omicron surge from December 16, 2021, to January 28, 2022. The median 1-10 rating during that time was 8 (IQR 4-10), and 139 (48.2%) users gave the tool a rating of 9 or 10. There was no difference in overall rating for responses during the Omicron surge compared with before or after the Omicron surge (*P*=.86).

### Demographics

Patient demographics of the survey respondents are summarized in Table 1. There were no significant differences in the user-reported care experience (“improved,” “made no impact,” or “worsened my care experience”) between cohorts by age, race, ethnicity, sex, or socioeconomic status (Table 2). A multivariate linear regression analysis similarly found no significant associations between user rating and respondent demographics and time frame of use. A subanalysis during the omicron peak found similar results.

**Table 1:** Patient demographics (N=395).

Variable		Value
Average rating, median (IQR)		8 (3-10)
**Age (years), n (%)**		
	18-39	45 (11.4)
	40-59	123 (31.1)
	>60	158 (40)
**Race, n (%)**		
	White	222 (56.2)
	Black or African American	11 (2.8)
	Asian, Native Hawaiian or Other Pacific Islander	51 (12.9)
	American Indian or Alaska Native	5 (1.3)
	Other or prefer not to answer	37 (9.4)
**Ethnicity, n (%)**		
	Hispanic	33 (8.4)
	Non-Hispanic	227 (57.5)
	Prefer not to answer or unknown	49 (12.4)
**Sex, n (%)**		
	Male	94 (23.8)
	Female	221 (55.9)
	Prefer not to answer	9 (2.3)
**Time frame, n (%)**		
	Omicron	288 (72.9)
	Before or after Omicron	107 (27.1)
Area deprivation index national percentile (IQR)		3 (2-9)

**Table 2:** Multivariable linear regression model to identify predictors of user rating.

Variable		Estimate	95% CI	*P* value	
**Age (vs 18-39)**					
	40-59	–0.65	–2.02, 0.72	.35	
	>60	0.13	–1.25, 1.52	.85	
Male (vs female)		0.08	–0.91, 1.08	.87	
Non-White (vs White)		0.79	–0.23, 1.82	.13	
Non-Hispanic (vs Hispanic)		–0.15	–1.58, 1.28	.84	
Area deprivation index		–0.02	–0.04, 0.00	.09	

### Alternatives to Care

**Figure 4:**
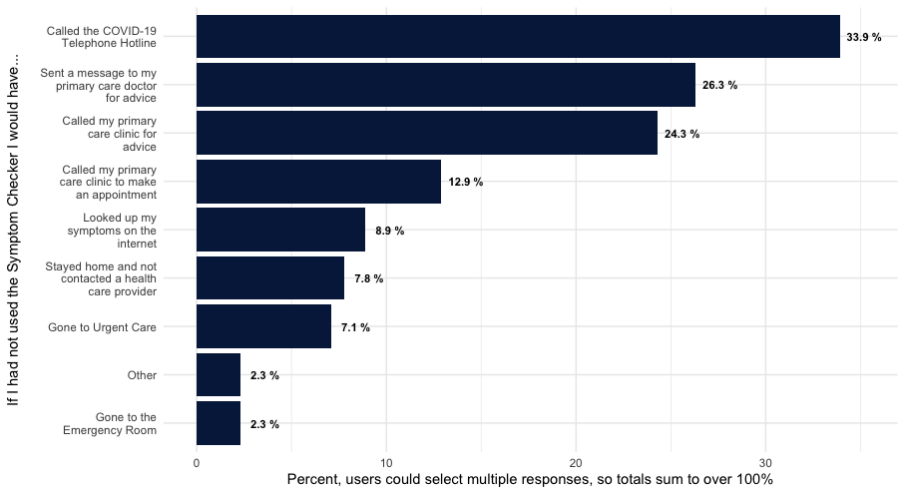
Patient-reported alternatives to COVID-19 Symptom Checker use (n=395).

Respondents gave a wide variety of answers when asked what they would have done if they did not have access to the web-based tool (Figure 4). The most common response was calling the COVID-19 telephone hotline (n=134, 33.9%), followed by sending a patient portal message to their physician (n=104, 26.3%) and calling their primary care clinic for advice (n=96, 24.3%).

### Open-ended Feedback

The most common categories of open-ended feedback were as follows: (1) requested changes to other COVID-19 services (eg, telephone hotline), policies, or procedures; (2) request for additional tool functionality; and (3) lack of appointment availability (Table 3). This feedback was used to inform updates and upgrades to the tool.

**Table 3:** Patient feedback categories, counts, and examples.

Category	Count	Examples and quotations
Requested changes to other COVID-19 services (eg, telephone hotline), policies, or procedures	24	“Should do covid testing 24 hours.” “Would be nice if you had MORE testing locations.” Long wait times when calling the hotline during surges Desire to be able to schedule preoperative or preadmission COVID-19 tests online
Request for additional functionality	19	“Would be good to see possible appointment times upfront and then opt to continue entering all one's personal info. Many people are first looking for an available time slot that can work for them.” Same-day cancelation or rescheduling of a visit online Ability for the tool to recall prior responses
Lack of appointment availability	12	Lack of availability of same- or next-day test appointments during Omicron surge “There are literally no appointments available even though it recommended I have one.”
Difficulty navigating patient portal	8	Confusion in differentiating this tool from a distinct, “schedule a visit” tool on the patient portal “I couldn't find the link to schedule a test. I began at ‘Schedule an appointment.’” Link to this tool not prominent enough
Request for more personalized health information	6	“How do I find out what ‘Your Value=Not Detected’ means? Does this mean I do not have Covid?” Request for quarantine advice personalized to individual circumstance
Outdated information or wording	6	Recommendation page listed an outdated clinic phone number
Difficult to understand care directions	4	“Didn’t go through with scheduling a visit/test because 1) it wasn’t clear if I had to have a video visit before the test; & 2) I didn’t know where I could get tested (for example, at primary care facility in ***?). So I aborted testing tool.”
Technical difficulties	4	“I did not get confirmation that the test was scheduled.” Network or connectivity problems

## Discussion

### Principal Findings

This study is the first, to our knowledge, to conduct user research on an EHR-integrated COVID-19 symptom checker with self-scheduling functionality. In this analysis, users generally perceived the symptom checker positively and usually reported that the tool improved their care experience. The most popular features were the ability to self-schedule a COVID-19 test online and 24-7 access to triage advice. Constructive or negative feedback on the tool was often a reflection of the larger ecosystem of care delivery for COVID-19, particularly during times of high demand, rather than related to the digital tool itself. This points toward the need for systems to develop robust, high-quality services in conjunction with usable and functioning digital health tools to aid in access.

### Comparison With Other Studies

This analysis supports existing literature [10,11,13] that COVID-19 symptom checkers are generally popular with users. It is well known that there are disparities in access to and ability to use digital tools [14-16]. However, we observed no differences in overall experience by age, sex, race, ethnicity, and socioeconomic status for those who successfully completed the tool. This suggests that disparities in the use and satisfaction of digital tools like this one may be primarily an issue of access to the tool itself, since those who successfully used the tool reported deriving an equivalent degree of benefit regardless of demographics. This finding can be extended to other patient portal–based tools such as self-scheduling and automated prescription refill services.

### Strengths and Weaknesses of the Study

This survey was completely optional, and we observed a low response rate, so there may be a risk for nonresponse bias. However, recent research suggests that the trends observed may accurately represent attitudes of the population despite low response rate [17,18]. Furthermore, we looked not only at quantitative trends but also at written feedback and noticed that most themes were mentioned in several responses, arguing that we may have reached saturation for likely responders or users with high digital literacy [19]. To keep the survey concise and anonymous, we were limited in the demographic information we could analyze and had no clinical information about the respondents. For that reason, we were unable to stratify the results based on clinical outcomes, comorbidities, or other patient factors. However, the anonymity of the survey likely promoted more open and honest feedback and responses. Finally, because we embedded the survey at the end of the tool, we were limited in our ability to assess technical barriers to tool use, since those who were unable to use the tool never saw the survey. However, we chose to locate the survey at the end of the tool because our primary goal was to receive timely and actionable user feedback. We chose not to conduct the survey by phone, email, or mail, out of concern that patients would confuse their Symptom Checker experience with other digital and telehealth tools (eg, video visits and remote monitoring) that they may use in the course of an illness, and because we wanted to avoid nonclinical patient communications during times of acute illness.

### Implications

This analysis may be useful to health systems that are trying to weigh the benefits of developing or integrating a COVID-19 symptom checker or similar tool with potential costs. In addition to the other established benefits, high patient satisfaction may make investment in COVID-19 symptom checkers worthwhile. Second, this tool may help to prevent front-line staff and physician burnout [20] by decreasing the volume of calls and patient portal messages. In total, this tool has been used over 80,000 times since it was introduced in March 2020. Based on the proportion of patients who responded that if they had not used this tool, they would have called or messaged their clinicians, the tool may have prevented over 20,000 calls and over 8800 patient messages to date. To our knowledge, this is the first study to assess patient alternatives to use of a symptom checker in the United States. It is consistent with literature from Switzerland and France that most users would have contacted health care systems in the absence of a self-triage tool, and that the use of these tools decreases call center volume [21,22]. Our results may also be generalizable to chatbots, which are widely used in health care to automate triage, connect patients, and reduce provider burden.

Several of the qualitative trends we observed may be useful to health systems and developers for the design and improvement of symptom checkers and other patient self-service tools. First, the most common category of written feedback was pertaining to the COVID-19 services, policies, and procedures, as well as appointment availability at the health system, rather than an intrinsic aspect of the tool. For that reason, it is essential that health systems first optimize their operational workflows and rightsize their capacity for tests and visits prior to or in conjunction with implementing such a tool. Second, users frequently requested software features that were beyond the current capabilities of the platform we used. Developers of these tools must therefore weigh the benefit of more nimble tools with user-friendly features (eg, transparency of visit availability) with the cost of their development and integration into the EHR. Third, users rarely reported technical challenges, suggesting usability of simple patient-portal–embedded tools. Fourth, the most popular feature of the symptom checker was the ability to self-schedule a COVID-19 test online, suggesting that EHR-integrated tools with the ability to offer self-scheduling of tests, screening exam, or imaging may be perceived more positively by patients compared with those without such features. Finally, our experience from reviewing continuous user feedback reinforced the notion that embedding a simple user feedback survey into a digital tool is a helpful way to promote iterative development.

### Conclusions

COVID-19 symptom checkers have effectively aided health systems in handling high volumes of triage and scheduling requests during the COVID-19 pandemic. We report high user satisfaction and user experience across demographic groups. Furthermore, patient-reported alternatives to the use of this tool suggest it may have saved thousands of phone calls and patient messages. COVID-19 symptom checkers are likely to remain in use for the near future in a diverse array of settings, and an examination of characteristics of use provides insight to improve the patient experience.

## Appendix

Multimedia Appendix 1 Qualtric survey questions embedded into electronic health record–integrated COVID-19 self scheduling and triage tool.

## Abbreviations

EHR: electronic health record
UCSF: University of California, San Francisco
