# Supplementary material for: Patient Experience and Feedback After Using an Electronic Health Record–Integrated COVID-19 Symptom Checker: Survey Study
Source: JMIR Hum Factors. 2022 Sep 13;9(3):e40064. doi: 10.2196/40064 (PMC9472505; doi:10.2196/40064)
Supplement: Supplementary file 1 [file humanfactors_v9i3e40064_app1.docx]

COVID Symptom Checker Feedback

Start of Block: Overall experience

Q1 How would you rate your overall experience using the UCSF MyChart COVID Testing and Care tool? (10 being the best, 0 being the worst)

|  | 0 | 1 | 2 | 3 | 4 | 5 | 6 | 7 | 8 | 9 | 10 |
| --- | --- | --- | --- | --- | --- | --- | --- | --- | --- | --- | --- |

| Experience with this MyChart tool () |  |
| --- | --- |

End of Block: Overall experience

Start of Block: Specific feedback

Q2 For each statement,
select the response that best reflects your experience with the UCSF MyChart COVID Testing and Care tool

|  | Strongly agree (1) | Somewhat agree (2) | Neither agree nor disagree (3) | Somewhat disagree (4) | Strongly disagree (5) |
| --- | --- | --- | --- | --- | --- |
| This tool was easy to use (1) |  |  |  |  |  |
| Questions were clear and easy to understand (2) |  |  |  |  |  |
| This tool helped me to get the care I needed (3) |  |  |  |  |  |

Q3 If I DID NOT have the UCSF COVID Testing and Care tool, I would have... (check all that apply)

- Sent a MyChart message to my primary care doctor for advice  (4) 

- Called the UCSF COVID-19 Telephone Hotline  (11) 

- Called my primary care clinic for advice  (1) 

- Called my primary care clinic to make an appointment  (2) 

- Gone to Urgent Care  (3) 

- Gone to the Emergency Room  (5) 

- Stayed home and not contacted a healthcare provider  (6) 

- Looked up my symptoms on the internet  (10) 

- Done something else (please specify below)  (8) ________________________________________________

Q4 The COVID Testing and Care tool...

- Improved my care experience  (1) 

- Made no impact on my care experience  (2) 

- Worsened my care experience  (3) 

Q5 Which features of the UCSF MyChart COVID Testing and Care tool are most important to you? (check all that apply)

- 24/7 access to triage advice if I have a symptom or possible COVID exposure  (1) 

- Ability to schedule my COVID test online  (5) 

- Ability to schedule my clinic or video visit appointment online, if recommended  (2) 

- Receiving COVID information and recommendations from UCSF clinical experts  (4) 

Q6 Did you have any technical difficulties using the MyChart COVID Testing and Care tool?

- Yes  (1) 

- No  (2) 

Display This Question:

If Did you have any technical difficulties using the MyChart COVID Testing and Care tool? = Yes

Q7 Which technical difficulties did you experience while using the MyChart COVID Testing and Care tool? (select all that apply)

- Trouble logging into MyChart  (1) 

- Difficulty finding the tool within MyChart  (2) 

- Hard time understanding certain questions  (3) 

- I was never given a care recommendation  (4) 

- Difficulty with the visit or test scheduling process  (5) 

- Technical glitch, like MyChart freezing, or not advancing to the next question  (8) 

- Other (please describe)  (7) ________________________________________________

End of Block: Specific feedback

Start of Block: Demographics

Q8 What is your age?

- 18-29 years  (1) 

- 30-39 years  (2) 

- 40-49 years  (3) 

- 50-59 years  (4) 

- 60-69 years  (5) 

- 70-79 years  (6) 

- 80 years or older  (7) 

Q9 What is your gender

- Male  (1) 

- Female  (2) 

- Genderqueer/Gender Non-binary  (3) 

- Trans Female  (4) 

- Trans Male  (5) 

- Not listed. Please specify  (6) ________________________________________________

- Prefer not to answer  (7) 

Q10 What is your ethnicity?

- Hispanic/Latino (LatinX)  (1) 

- Not hispanic  (2) 

- Unknown  (4) 

- Prefer not to answer  (5) 

Q11 What is your race? (select all that apply)

- Asian  (1) 

- American Indian or Alaska Native  (3) 

- Black or African American  (2) 

- Middle Eastern/West Asian or North African  (5) 

- Native Hawaiian or Pacific Islander  (6) 

- White  (7) 

- Other  (8) 

- Prefer not to answer  (9) 

End of Block: Demographics

Start of Block: Wrap up

Q12 If you have any other comments or suggestions about the UCSF MyChart COVID Testing and Care tool please leave them here: 

________________________________________________________________

End of Block: Wrap up
